# Clinical characteristics of obese, fixed airway obstruction, exacerbation-prone phenotype and comorbidities among severe asthma patients: a single-center study

**DOI:** 10.1186/s12890-023-02835-4

**Published:** 2024-02-09

**Authors:** Guiping Zhu, Yuqing Mo, Ling Ye, Hui Cai, Yingying Zeng, Mengchan Zhu, Wenjun Peng, Xin Gao, Xixi Song, Chengyu Yang, Jian Wang, Zhihong Chen, Meiling Jin

**Affiliations:** 1 Department of Pulmonary and Critical Care Medicine, Zhongshan Hospital, Fudan University, 200032 Shanghai, China; 2 Department of Allergy, Zhongshan Hospital, Fudan University, 200032 Shanghai, China; 3 The Affiliated Cancer Hospital of Zhengzhou University & Henan Cancer Hospital, 450008 Zhengzhou, China

**Keywords:** Severe asthma, Comorbidities, Exacerbations, Overweight or obesity, Fixed airway obstruction

## Abstract

**Background:**

Severe asthma places a large burden on patients and society. The characteristics of patients with severe asthma in the Chinese population remain unclear.

**Methods:**

A retrospective review was conducted in patients with severe asthma. Demographic and clinical data were collected. Patients were grouped according to phenotypes in terms of exacerbations, body mass index (BMI) and fixed airway obstruction (FAO) status, and the characteristics of different groups were compared. Comorbidities, factors that influence asthma phenotypes, were also analyzed in the study.

**Results:**

A total of 228 patients with severe asthma were included in our study. They were more likely to be overweight or obese. A total of 41.7% of the patients received GINA step 5 therapy, and 43.4% had a history of receiving regular or intermittent oral corticosteroids (OCS). Severe asthmatic patients with comorbidities were prone to have more asthma symptoms and decreased quality of life than patients without comorbidities. Patients with exacerbations were characterized by longer duration of asthma, poorer lung function, and worse asthma control. Overweight or obese patients tended to have more asthma symptoms, poorer lung function and more asthma-related comorbidities. Compared to patients without FAO, those in the FAO group were older, with longer duration of asthma and more exacerbations.

**Conclusion:**

The existence of comorbidities in patients with severe asthma could result in more asthma symptoms and decreased quality of life. Patients with exacerbations or with overweight or obese phenotypes were characterized by poorer lung function and worse asthma control. Patients with FAO phenotype tended to have more exacerbations.

## Introduction

Severe asthma is defined as requiring Global Initiative for Asthma (GINA) step 4/5 interventions to prevent it from becoming “uncontrolled”, or remaining “uncontrolled” despite this treatment. It accounts for approximately 5–10% of all patients with asthma [1]. The China Asthma and Risk Factors Epidemiological Survey (CARE) showed that the prevalence of asthma in Chinese adults was 4.2%, of which severe asthma accounted for 5.99% [2]. Severe asthma places a large physical, mental, emotional, social, and economic burden on patients, and is associated with major treatment and socioeconomic burdens of a country [3, 4].

Compared to individuals with mild-to-moderate asthma, those with severe asthma have been reported to have more symptoms, exacerbations, and comorbidities [5]. In particular, various phenotypes of severe asthma have been proposed based on different clinical characteristics such as age at the onset of asthma, airway inflammation, atopic status and other characteristics [6, 7]. According to the Guidelines for Bronchial Asthma Prevent and Management in China, severe asthma was divided into 5 clinical phenotypes: early-onset allergic asthma; late-onset persistent eosinophilic asthma; asthma with frequent exacerbations; obese asthma; and asthma with fixed airway obstruction [8]. Acute exacerbations remain a serious challenge in asthma treatment and an explicit cause of progressive loss of lung function in asthmatic patients [9, 10]. It is defined as a new phenotype of asthma named exacerbation-prone asthma (EPA) [11]. Obese asthma is considered as a complex syndrome, as well as another phenotype of asthma [12]. The incidence of obesity in asthma patients varies from country to country [13, 14], with nearly 40% of severe asthma patients being obese around the world [15]. Obese asthmatic patients tend to have more symptoms, more frequent and severe exacerbations, and decreased quality of life [16]. Fixed airflow obstruction (FAO) is another feature of severe asthma [17] and presented as a new phenotype [18].Comorbidities are important in the management of severe asthma, and contribute to poor disease control by aggravating symptoms through affecting severe asthma phenotype and treatment response [19, 20].

Identifying the clinical characteristics of different phenotypes and recognizing comorbidities is a strategic approach in the treatment of severe asthma and could help to predict patients at high risk of exacerbation, formulating optimal individualized therapeutic schedule, and achieving effective asthma control [21]. Since the characteristics of severe asthma patients in the Chinese population remain unclear, in this study, we retrospectively analyzed the clinical characteristics of obese, fixed airway obstruction, exacerbation-prone phenotype and comorbidities among severe asthma patients. The results of this study will improve our understanding of severe asthma in the Chinese population, and thus, identify differences in the clinical characteristics of different phenotypes.

## Materials and methods

### Study design and setting

We screened all patients diagnosed with severe asthma from the asthma database of Zhongshan hospital between January 2016 and December 2020. Severe asthma is defined as requiring Global Initiative for Asthma (GINA) step 4/5 to prevent it from becoming “uncontrolled”, or remaining “uncontrolled” despite this treatment [1, 8]. This study was approved by the ethics committee of Zhongshan hospital, Fudan university (approval number: B2019-020R), and was conducted in accordance with the Declaration of Helsinki.

### Patients

Patients in our study were 14 years or older. They received treatment with medium-to-high dose inhaled corticosteroid (ICS) and long-acting beta agonist (LABA) with or without other controller medications. Patients with allergic bronchopulmonary aspergillosis (ABPA), chronic obstructive pulmonary disease (COPD), and chronic respiratory failure with long-term use of non-invasive positive pressure ventilation were excluded.

### Data collection and definition

Demographic and clinical data including age, sex, height, weight, smoking status, lung function, FENO, and allergen detection results were recorded. Symptom control was assessed using the Asthma Control Test (ACT), and the score ranged from 5 to 25, with a higher score reflecting better asthma control. Quality of life was measured with the Asthma Quality of Life Questionnaire (AQLQ).

Both regular and intermittent oral corticosteroid (OCS) use were included in OCS use. Regular OCS use is defined as a prescription for ≥ 90 days of OCS exposure in the previous year. Intermittent OCS use is defined as a prescription for repeated OCS use and/or ≥ 2 exacerbations (treated with OCS) [15]. The number of exacerbations was defined as the number that required the use of corticosteroids for at least 3 days or asthma-related hospitalization or emergency visit [22] in the previous year. Comorbidities were identified by clinical symptoms supported by the associated validated screening questionnaires for allergic rhinitis (the score for allergic rhinitis value ≥ 7) [23], chronic rhino-sinusitis (sinonasal questionnaire score ≥ 1) [24], obstructive sleep apnea (Berlin questionnaire score ≥ 2) [25], gastroesophageal reflux disease (GERD questionnaire score > 2) [26], anxiety/depression (hospital anxiety and depression score ≥ 11) [27], dysfunctional breathing (Nijmegen score ≥ 23) [28], and vocal cord dysfunction (Pittsburgh vocal cord dysfunction index ≥ 4) [29] as described in previous study [30]. The Chinese-specific cut-off points of body mass index (BMI) was categorized as follows: (1)obesity as BMI ≥ 28 kg/m^2^, (2)overweight as BMI 24.0-27.9 kg/m^2^, (3)normal as BMI 18.5–23.9 kg/m^2^, (4)underweight as BMI < 18.5 kg/m^2^ [31]. Allergen-specific immunoglobulin E (sIgE) was tested for house dust mite (hx2), molds and yeasts mix (mx2), food allergen mix (fx5), weed pollen mix (wx5) and tree pollen mix (tx4).

The smoking status of patients was included in our study. Never smokers were defined as individuals who had never smoked regularly or smoked less than 100 cigarettes during their lifetime. Ex-smokers were defined as individuals who had previously smoked more than one cigarette each day but had quit smoking for more than 1 year. Current smokers were defined as persons who smoked more than one cigarette per day and had smoked for more than 1 year [32]. Heavy smokers were defined as smoking > 10 PY, indicating smoking one pack (20 cigarettes) daily for 10 years [33, 34].Heavy smokers were excluded in the study.

FAO was defined as a persistent status of the ratio of forced expiratory volume to forced vital capacity in 1 s (FEV_1_/FVC) below 0.70 after bronchodilator inhalation [35]. COPD is a heterogeneous lung condition characterized by chronic respiratory symptoms due to abnormalities of the airways and/or alveoli that cause persistent, often progressive airflow obstruction [36].

The clinical features of different phenotypes were analyzed in terms of exacerbations, BMI and FAO. Since this was a retrospective study to investigate the clinical characteristics of severe asthma in China, the data are somewhat incomplete. Therefore, we divided the available data into different subgroups for analysis, expecting more valuable results.

### Statistical analysis

Statistical analysis was performed with SPSS (version 23.0; IBM Corporation, Armonk, New York). Normal distribution variables were shown as mean ± SD and compared using Student’s t-test or one-way ANOVA test. Non-normally distributed variables were shown as median with interquartile range and compared using Mann-Whiteny test or Kruskal-Wallis test. Categorical data were shown as frequencies with percentages and compared using chi-squared or Fisher’s exact test. Linear regression was used to investigate factors associated with quality of life. Multivariate logistic regression analysis was conducted to asses factors related to uncontrolled asthma. A *p*-value < 0.05 was considered to be statistically significant.

## Results

### Demographic and clinical characteristics of patients

**Fig. 1:**
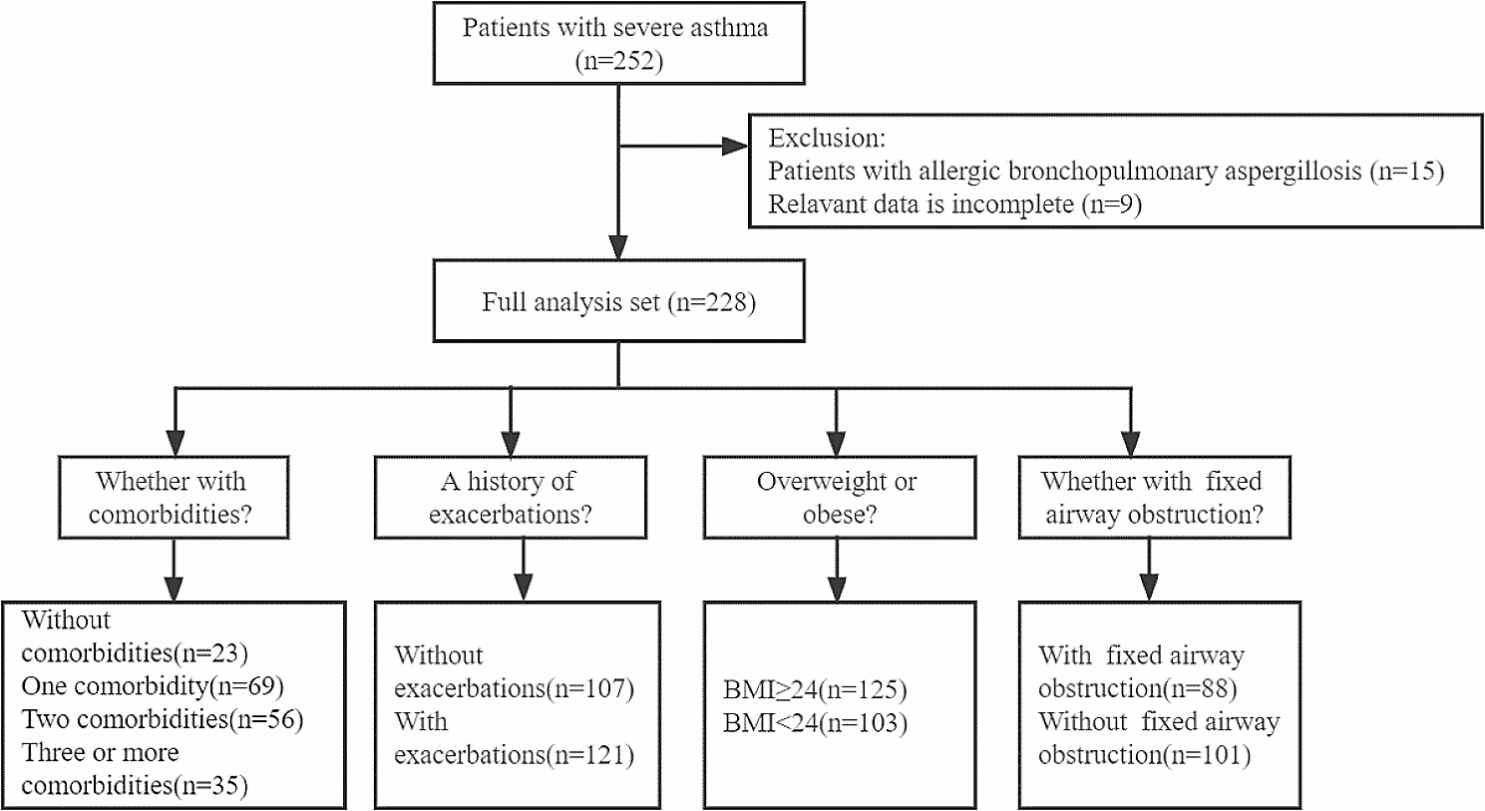
Flow diagram of study participants

A flow chart of the research was shown in Fig. 1. This study included 228 patients with severe asthma. The demographic and clinical characteristics of enrolled patients were described in Table 1. The median age of all patients was 53.0 (38.0–62.0) years and median age at asthma onset was 40.0 (22.3–50.8) years. Patients were mainly male (52.6%), overweight or obese (54.8%), and never smoked (74.6%). More than half of the patients (66.3%) were allergen-positive. Allergic rhinitis was the predominant comorbidity (76.7%) in the total population, followed by gastroesophageal reflux (40.3%). Ninety-five (41.7%) patients received treatment at GINA step 5. A total of 43.4% of the patients had a history of receiving regular or intermittent OCS, and 80 (35.1%) patients were receiving biologics (Omalizumab).

**Table 1:** Basic characteristics of patients included in the study

Characteristic (n = 228)	data
Sex, No. (%)	
Female	108 (47.4%)
Male	120 (52.6%)
Age (years)	53.0 (38.0–62.0)
Age of diagnosis (years)	40.0 (22.3–50.8)
Duration of asthma	7.0 (4.0–19.0)
BMI, kg/m^2^	
Underweight (< 18.5)	8 (3.5%)
Normal (≥ 18.5 to < 24)	95 (41.7%)
Overweight (≥ 24 to < 28)	90 (39.5%)
Obesity (≥ 28)	35 (15.4%)
Smoking status	
Current smoker	15 (6.6%)
Ex-smoker	43 (18.9%)
Never smoked	170 (74.6%)
Allergen-specific IgE (n = 181)	
positive hx2-positive	120 (66.3%) 96 (53.0%)
mx2-positive	34 (18.8%)
fx5-positive	20 (11.0%)
wx5-positive	16 (8.8%)
tx4-positive	14 (7.7%)
negative	61 (33.7%)
GINA classification	
Step 4	133 (58.3%)
Step 5	95 (41.7%)
Laboratory findings	
Blood neutrophil (%) (n = 159)	57.3 (50.7–64.9)
Blood neutrophil (/ul) (n = 157)	4000.0 (3300.0-5300.0)
Blood eosinophil (%) (n = 172)	3.6 (1.7-8.0)
Blood eosinophil (/ul) (n = 168)	235.0 (130.0-550.0)
Total IgE, IU/ml (n = 186)	250.0 (96.1-517.8)
Comorbidities	
Allergic rhinitis (n = 176)	135 (76.7%)
Chronic sinusitis (n = 111)	34 (30.6%)
Gastroesophageal reflux (n = 139)	56 (40.3%)
Obstruction sleep apnea (n = 96)	17 (17.7%)
Anxiety/Depression (n = 104)	32 (30.8%)
Vocal cord dysfunction (n = 100)	33 (33.0%)
Dysfunctional breathing (n = 98)	9 (9.2%)
Asthma-related medications other than ICS/LABA	
LAMA	39 (17.1%)
LTRA	118 (51.8%)
Theophylline	54 (23.7%)
Omalizumab	80 (35.1%)
OCS use	99 (43.4%)
Bronchial thermoplasty	3 (1.3%)

Values are presented as number (%) and mean ± SD or median with interquartile range

Abbreviations: BMI, body mass index; hx2, house dusts mix; mx2, molds and yeasts mix; fx5, food allergen mix; wx5, weed pollen mix; tx4, tree pollen mix;IgE, immunoglobulin E; ICS, inhaled corticosteroid; LABA, long-acting beta agonist; LAMA, long-acting muscarine antagonist; LTRA, leukotriene receptor antagonist; OCS, oral corticosteroid

### Comparison of characteristics of patients according to comorbidities

A total of 183 patients were included in this subgroup analysis. As shown in Table 2, the characteristics of patients according to comorbidities were analyzed. Patients with 3 or more comorbidities had lower ACT and AQLQ scores than those with less than 3 or without comorbidities (*P* = 0.004; *P* = 0.007). 60% of patients with 3 or more comorbidities received treatment at GINA step 5, which was the highest compared to other groups (*P* = 0.039). The requirement of the use of corticosteroids for at least 3 days during exacerbations was more likely to occur when patients had more comorbidities (*P* = 0.013). There were no significant differences in age, lung function, neutrophils, eosinophils, or total IgE levels among the groups.

**Table 2:** Comparison of characteristics of patients according to comorbidities

	Comorbidities (0) (n = 23)	Comorbidities (1) (n = 69)	Comorbidities(2) (n = 56)	Comorbidities(≥3) (n = 35)	*P* Value
Age (years)	53.0 (40.0–59.0)	48.0 (33.5–61.5)	51.5 (37.5–59.5)	50.0 (37.0–63.0)	0.858
Sex, female	13 (56.5%)	31 (44.9%)	16 (28.6%)	18 (51.4%)	0.765
Age of diagnosis (years)	45.0 (20.0–54.0)	34.0 (15.0–50.0)	35.5 (20.0–48.0)	33.0 (20.0–46.0)	0.350
Duration of asthma	8.0 (3.0–17.0)	8.0 (4.0-19.5)	7.5 (4.0–20.0)	7.0 (5.0–25.0)	0.802
Pre-bronchodilatorFEV_1_% pred (n = 175)	73.7 ± 25.1	68.8 ± 23.7	69.8 ± 23.9	66.2 ± 25.7	0.723
Pre-bronchodilator FEV_1_/FVC(%) (n = 177)	68.4 ± 14.3	65.8 ± 14.7	64.2 ± 14.4	63.3 ± 15.3	0.575
Post-bronchodilator FEV_1_% pred (n = 152)	76.0 ± 25.3	77.7 ± 21.5	77.2 ± 23.2	70.7 ± 24.7	0.593
Post-bronchodilator FEV_1_/FVC(%) (n = 157)	71.6 (61.4–80.8)	70.7 (62.5–81.4)	70.6 (60.0-80.2)	64.8 (60.7–83.1)	0.764
FENO, ppb (n = 128)	58.0 (19.0-99.5)	45.5 (26.5–63.8)	43.0 (23.0–95.0)	31.0 (17.3–48.0)	0.201
Blood neutrophil (%) (n = 125)	61.7 (52.3–68.9)	57.1 (50.8–68.3)	57.3 (50.9–64.3)	59.9 (52.4–64.1)	0.764
Blood neutrophil (/ul) (n = 123)	4700.0(3675.0-5825.0)	4000.0 (3325.0-5425.0)	4000.0 (3190.0-4975.0)	4400.0 (3220.0-6800.0)	0.339
Blood eosinophil (%) (n = 138)	3.9 (1.0-8.5)	3.5 (2.0–8.0)	4.0 (1.8–8.2)	2.9 (1.1–5.7)	0.669
Blood eosinophil (/ul) (n = 134)	240.0 (80.0-500.0)	235.0(150.0-600.0)	245.0 (110.0-550.0)	150.0(70.0-355.0)	0.445
Total IgE, IU/ml (n = 148)	219.0 (124.3-658.3)	272.0 (92.4–724.0)	272.0 (109.0-636.0)	191.0 (120.0-403.0)	0.825
ACT score (n = 164)	21.0 (18.5–23.0)	20.5 (19.0–23.0)	20.0 (19.0–22.0)	18.0(16.0–21.0)	0.004
AQLQ score (n = 122)	5.9 (5.5–6.2)	4.7 (3.2–5.9)	4.6 (3.7–5.6)	4.0 (2.9–5.2)	0.007
GINA step 5	13 (56.5%)	29 (42.0%)	18 (32.1%)	21 (60.0%)	0.039
Number of exacerbations	0 (0–2)	0 (0–2)	1 (0–2)	2 (0–3)	0.102
Corticosteroids treatment for at least 3 days	7 (30.4%)	20 (29.0%)	25 (44.6%)	21 (60.0%)	0.013
Emergency department visit or hospitalization	3 (13.0%)	15 (21.7%)	11 (19.6%)	5 (14.3%)	0.791

Values are presented as number (%) and mean ± SD or median with interquartile range

Abbreviations: FEV_1_, forced expiratory volume in 1 s; FVC, forced vital capacity; FENO, fractional exhaled nitric oxide; IgE, immunoglobulin E; ACT, asthma control test; AQLQ, asthma quality of life questionnaire

### Comparison of characteristics between patients with and without exacerbations in the previous year

Sex, age at asthma onset, laboratory findings, and number of comorbidities were similar between the two groups. Compared to patients without exacerbations, the average age of patients with exacerbations was older and the duration of asthma was longer. Exacerbations were related to progressive loss of lung function in asthmatic patients. In this study, poorer lung function was observed in patients with exacerbations, regardless of pre-bronchodilator or post-bronchodilator. Furthermore, patients had better asthma control and higher quality of life if they did not experience exacerbations. Compared to those without exacerbations, patients with exacerbations had a higher percentage of receiving treatment at GINA step 5 (Table 3).

**Table 3:** Comparison of characteristics between patients with and without exacerbations

	Exacerbations ≥ 1 (n = 121)	No Exacerbations (n = 107)	*P* Value
Age (years)	55.0 (43.0–63.0)	48.0 (37.0–59.0)	0.003
Sex, female	56 (46.3%)	52 (48.6%)	0.158
Age of diagnosis (years)	41.0 (21.0–52.0)	37.0 (22.5–50.0)	0.757
Duration of asthma	9.0 (5.0–22.0)	6.0 (3.0–15.0)	0.005
Pre-bronchodilator FEV_1_% pred(n = 219)	61.9 ± 23.5	74.9 ± 22.9	< 0.001
Pre-bronchodilator FEV_1_/FVC (%) (n = 221)	60.2 ± 13.7	69.1 ± 13.9	< 0.001
Post-bronchodilator FEV_1_% pred (n = 184)	70.3 ± 22.6	80.8 ± 22.2	0.002
Post-bronchodilator FEV_1_/FVC (%) (n = 189)	65.1 (59.5–75.2)	73.5 (64.0-83.5)	< 0.001
FENO, ppb (n = 161)	41.0 (23.8–73.8)	40.0 (24.0–83.0)	0.874
Blood neutrophil (%) (n = 159)	57.7 (50.9–66.3)	56.2 (50.7–62.4)	0.325
Blood neutrophil (/ul) (n = 157)	4100.0 (3400.0-5500.0)	3900.0 (3225.0-5175.0)	0.325
Blood eosinophil (%) (n = 172)	3.1 (1.5–6.3)	4.6 (2.1–8.5)	0.079
Blood eosinophil (/ul) (n = 168)	205.0 (122.0-367.5)	315.0 (140.0-632.5)	0.044
Total IgE, IU/ml (n = 186)	202.0 (74.2–405.0)	282.0 (128.0-533.0)	0.075
ACT score (n = 198)	19.0 (18.0–21.0)	21.0 (19.0–23.0)	0.001
AQLQ score (n = 130)	4.2 ± 1.2	4.9 ± 1.3	0.001
GINA step 5	56 (46.3%)	40 (37.4%)	0.003
Number of comorbidities (n = 183)			0.116
0	10 (10.5%)	13 (14.8%)	
1	30 (31.6%)	39 (44.3%)	
2	32 (33.7%)	24 (27.3%)	
≥ 3	23 (24.2%)	12 (13.6%)	

Values are presented as number (%) and mean ± SD or median with interquartile range

Abbreviations: FEV_1_, forced expiratory volume in 1 s; FVC, forced vital capacity; FENO, fractional exhaled nitric oxide; IgE, immunoglobulin E; ACT, asthma control test; AQLQ, asthma quality of life questionnaire

### Comparison of characteristics between patients with BMI ≥ 24 and BMI < 24

To understand the role of overweight or obesity in severe asthma, 228 patients were divided into two groups with BMI ≥ 24 or BMI < 24 and the characteristics were analyzed in Table 4. There were no differences in age, age at asthma onset, duration of asthma, or laboratory findings. The percentage of female in BMI ≥ 24 group was lower than in BMI < 24 group (*P* = 0.007). Compared to BMI < 24 group, the measurement of the percentage of forced expiratory volume in 1 s (FEV_1_% pred) after bronchodilator inhalation was lower in BMI ≥ 24 group (*P* = 0.011). The BMI ≥ 24 group had worse asthma control as measured by ACT than the BMI < 24 group (*P* = 0.007). The percentage of patients with 3 or more comorbidities in BMI ≥ 24 group was 26.5%, much higher than BMI < 24 group.

**Table 4:** Comparison of characteristics between patients with BMI ≥ 24 and BMI < 24

	BMI ≥ 24 (n = 125)	BMI < 24 (n = 103)	*P* Value
Age (years)	53.0 (40.5–62.0)	50.0 (36.0–63.0)	0.194
Sex, female	49 (39.2%)	59 (57.3%)	0.007
Age of diagnosis (years)	41.0 (20.0-50.5)	37.0 (23.0–51.0)	0.838
Duration of asthma	8.0 (4.0–20.0)	6.0 (3.0–15.0)	0.036
Pre-bronchodilator FEV_1_% pred (n = 219)	66.0 ± 22.5	72.3 ± 25.4	0.055
Pre-bronchodilator FEV_1_/FVC (%) (n = 221)	64.1 ± 12.9	65.8 ± 16.3	0.408
Post-bronchodilator FEV_1_% pred (n = 184)	72.1 ± 22.3	80.7 ± 22.9	0.011
Post-bronchodilator FEV_1_/FVC (%) (n = 189)	68.2 (61.5–78.3)	71.8 (61.8–83.4)	0.105
FENO, ppb (n = 161)	37.5 (23.8–65.8)	48.0 (26.0–95.0)	0.169
Blood neutrophil (%) (n = 159)	57.3 (50.8–62.9)	57.1(50.7–66.1)	0.799
Blood neutrophil (/ul) (n = 157)	4100.0(3300.0-5500.0)	3900.0 (3300.0-5200.0)	0.630
Blood eosinophil (%) (n = 172)	3.9 (2.1–8.2)	3.4 (1.5–7.6)	0.420
Blood eosinophil (/ul) (n = 168)	235.0 (140.0-572.5)	235.0 (110.0-530.5)	0.514
Total IgE, IU/ml (n = 186)	227.0 (96.6-564.5)	272.0 (86.6-491.5)	0.986
ACT score (n = 198)	20.0 (17.0–21.0)	21.0 (19.0–23.0)	0.007
AQLQ score (n = 130)	4.4 (3.5–5.4)	4.7 (3.4–5.8)	0.521
GINA step 5	54 (43.2%)	42 (40.8%)	0.712
Number of exacerbations	0 (0–2)	0 (0–2)	0.568
Corticosteroids treatment for at least 3 days	47 (37.6%)	37 (35.9%)	0.794
Emergency department visit or hospitalization	23 (18.4%)	17 (16.5%)	0.687
Number of comorbidities (n = 183)			0.018
0	10 (10.2%)	13 (15.3%)	
1	30 (30.6%)	39 (45.9%)	
2	32 (32.7%)	24 (28.2%)	
≥ 3	26 (26.5%)	9 (10.6%)	

Values are presented as number (%) and mean ± SD or median with interquartile range

Abbreviations: FEV_1_, forced expiratory volume in 1 s; FVC, forced vital capacity; FENO, fractional exhaled nitric oxide; ACT, asthma control test; AQLQ, asthma quality of life questionnaire

Considering that there were significant differences in other covariates such as sex, ACT score, and comorbidities according to BMI, multiple linear regression analysis was performed to evaluate the effects of BMI, sex, number of comorbidities, and ACT scores on post-bronchodilator FEV_1_% pred. As shown in Table 5, there was a negative correlation between lung function and BMI(P = 0.022), while other factors did not significantly affect lung function.

**Table 5:** Multiple linear regression analysis of factors associated with post-bronchodilator FEV_1_% pred

	Β (95%CI)	*t* value	*P* value
Sex, female	-0.562(-8.303,7.179)	-0.144	0.886
BMI	-1.318(-2.447, -0.190)	-2.312	0.022
Number of comorbidities	-0.031(-4.306, 4.245)	-0.014	0.989
ACT score	0.104(-0.997, 1.205)	0.187	0.852

### Comparison of characteristics between patients with and without FAO

In order to further clarify the clinical characteristics of patients with FAO, we divided the patients into FAO and non-FAO groups (Table 6). Eighty-eight (46.6%) patients had FAO. Sex, BMI, and age at asthma onset were similar between the two groups. Patients in FAO group were older and had longer duration of asthma than those in non-FAO group. Compared to non-FAO group, FEV_1_% pred was lower in FAO group. The value of FENO, laboratory findings, asthma control, and quality of life did not differ significantly between two groups. However, the exacerbations of asthma and the percentage of patients requiring the use of corticosteroids for at least 3 days were affected by FAO (*P* = 0.004; *P* = 0.007). The percentage of severe asthma at GINA step 5 (65.9%) in FAO group was significantly higher compared to non-FAO group.

**Table 6:** Comparison of characteristics between patients with and without FAO

	FAO (n = 88)	Non-FAO (n = 101)	*P* Value
Age (years)	56.0 (47.0–63.0)	48.0 (32.5–59.0)	< 0.001
Sex, female	43 (48.9%)	48 (47.5%)	0.854
Age of diagnosis (years)	41.5 (24.3–49.8)	36.0 (20.0–51.0)	0.846
Duration of asthma	11.0 (5.0-23.5)	5.0 (2.0-12.5)	< 0.001
Pre-bronchodilator FEV_1_% pred (n = 185)	51.2 ± 15.2	84.1 ± 17.8	< 0.001
Pre-bronchodilator FEV_1_/FVC (%) (n = 187)	54.9 ± 8.6	74.8 ± 10.5	< 0.001
Post-bronchodilator FEV_1_% pred (n = 184)	57.9 ± 16.4	91.2 ± 15.2	< 0.001
Post-bronchodilator FEV_1_/FVC (%) (n = 189)	61.3 (55.3–64.9)	79.1 (73.2–84.5)	< 0.001
FENO, ppb (n = 135)	39.5 (22.0-78.5)	41.0 (27.0–82.0)	0.440
Blood neutrophil (%) (n = 131)	59.3 (51.7–65.6)	56.5 (50.3–62.8)	0.319
Blood neutrophil(/ul) (n = 130)	4100.0 (3150.0-5700.0)	3900.0 (3300.0-4800.0)	0.474
Blood eosinophil (%) (n = 143)	3.7 (1.5-8.0)	4.2 (2.0-8.7)	0.440
Blood eosinophil (/ul) (n = 141)	230.0 (100.0-585.0)	300.0 (140.0-595.0)	0.230
Total IgE, IU/ml (n = 153)	183.5 (75.5-396.8)	272.0 (129.5-521.5)	0.082
ACT score (n = 161)	19.5 ± 3.6	20.0 ± 3.7	0.321
AQLQ score (n = 105)	4.5 ± 1.3	4.6 ± 1.4	0.742
GINA step 5	58 (65.9%)	32 (31.7%)	< 0.001
Number of exacerbations	1 (0–2)	0 (0–2)	0.004
Corticosteroids treatment for at least 3 days	42 (47.7%)	29 (28.7%)	0.007
Emergency department visit or Hospitalization	20 (22.7%)	13 (12.9%)	0.075
Number of comorbidities (n = 157)			0.733
0	7 (9.5%)	11 (13.3%)	
1	26 (35.1%)	31 (37.3%)	
2	24 (32.4%)	27 (32.5%)	
≥ 3	17 (23.0%)	14 (16.9%)	

Values are presented as number (%) and mean ± SD or median with interquartile range

Abbreviations: FAO, fixed airway obstruction; FEV_1_, forced expiratory volume in 1 s; FVC, forced vital capacity; FENO, fractional exhaled nitric oxide; IgE, immunoglobulin E; ACT, asthma control test; AQLQ, asthma quality of life questionnaire

### Multiple regression analysis for factors associated with quality of life and Asthma control

Factors like age, sex, number of exacerbations, number of comorbidities, duration of asthma, and pre-bronchodilator FEV_1_% pred were taken into analysis after univariate analysis. As shown in Table 7, exacerbations and comorbidities were significantly linked to patients’ quality of life in the multiple linear regression. Patients with more exacerbations or comorbidities tended to have decreased quality of life. In addition, female patients had lower quality of life than male. The older the patients were, the more it affected their life.

**Table 7:** Linear regression for factors associated with quality of life

	B (95% CI)	t value	*P* value
Age	-0.018 (-0.035, -0.001)	-2.099	0.038
Sex, female	-0.637 (-1.077, -0.198)	-2.876	0.005
Duration of asthma	-0.010 (-0.024, 0.004)	-1.383	0.170
Number of exacerbations	-0.152 (-2.252, 0.053)	-3.033	0.003
Pre-bronchodilator FEV_1_% pred	0.003 (-0.007, 0.013)	0.585	0.560
Number of comorbidities	-0.292 (-0.531, -0.052)	-2.418	0.017

FEV_1_, forced expiratory volume in 1 s; FVC, forced vital capacity

Five potential risk factors of age, duration of asthma, number of exacerbations, sex, and number of comorbidities were included in logistic regression analysis after the univariate analysis. The logistic regression analysis showed that more exacerbations were associated with higher odds (OR = 1.229, *P* = 0.025). Similarly, more than 3 comorbidities were associated with higher odds (OR = 5.494, *P* = 0.009). Besides, female patients were more likely to have poorer asthma control than male (OR = 0.406, *P* = 0.013). (Table 8).

**Table 8:** logistic regression analysis of uncontrolled asthma (ACT < 20)

	OR (95% CI)	*P* value
Age	1.011(0.987, 1.035)	0.380
Duration of asthma	1.019 (0.995, 1.045)	0.126
Number of exacerbations	1.229 (1.210, 5.012)	0.025
Sex, female	2.462(0.200, 0.827)	0.013
Number of comorbidities		0.014
1	1.665 (0.520, 5.327)	0.390
2	1.232 (0.381, 3.988)	0.728
≥3	5.494 (1.531, 19.718)	0.009

## Discussion

In our present study, patients with severe asthma were mainly overweight or obese, allergen-positive and never smoked. Patients enrolled in our study had a severe disease status, as a total of 41.7% of the patients were receiving GINA step 5 therapy, and 43.4% had a history of receiving regular or intermittent OCS. Approximately 90% of patients had asthma-related comorbidities and 53.1% of them experienced at least one exacerbation in the previous year. Approximately half of patients in this cohort had fixed airway obstruction.

The most common comorbidity in patients with severe asthma in our study was allergic rhinitis, and almost half of patients had gastroesophageal reflux. Asthma and allergic rhinitis have similar triggers and pathophysiology, characterized by similar inflammatory cell infiltrates [37]. Previous studies showed that patients with allergic rhinitis had distinct abnormalities of lower airway function although they had not yet developed into asthma [38, 39]. The relationship between asthma and gastroesophageal reflux is bidirectional. Acid infusion into the esophagus may cause bronchoconstriction, on the other hand, asthma may predispose to gastroesophageal reflux [40]. Gastroesophageal reflux is associated with frequent asthma exacerbations and oral corticosteroid therapy during exacerbations [41]. More than a quarter of patients had anxiety/depression, which may result in poorer quality of life and asthma control [42]. Strong associations were detected between comorbidities and severe asthma [43]. Comorbidities are important to the increased risk and frequency of annual hospitalizations due to asthma exacerbation [44]. In accordance with our present results, patients with 3 or more comorbidities had more symptoms and decreased quality of life. Comorbidities also increased the likelihood of systemic corticosteroids use when exacerbation. Therefore, recognition of these comorbidities is important for asthma management.

There are no precise biomarkers for the recognition of patients who are prone to exacerbations of asthma. A cross sectional study found that blood and sputum eosinophils, FEV_1_, depression, and some other factors are high risk triggers of asthma symptoms [45]. A meta-analysis of 23 observational studies validated that blood eosinophil counts ≥ 200 cells/µL were associated with asthma exacerbations [46]. Contrary to this finding, blood eosinophils were lower in patients with exacerbations in our study, which may be related to neutrophilic inflammation caused by rhinovirus. Rhinoviruses are known to be important triggers of asthma exacerbations [47, 48]. Lung function in patients with exacerbations was worse than in patients without exacerbations, further identifying the finding that exacerbations are the main cause of progressive loss of lung function [9]. Besides, poor lung function was also considered to be an independent factor of a higher probability of hospital readmission [49], forming a vicious circle between poor lung function and frequent exacerbations.

Asthma is usually controlled with inhaled corticosteroids. Obese asthmatics do not respond as well to standard controller medications such as ICS and combination ICS-long-acting beta agonists (LABA) [50]. Obese asthma may represent a unique phenotype of asthma, with a more severe disease outcome due to impairment of efficacy of conventional therapy [51]. As shown in our study, overweight or obese asthmatic patients had poorer asthma control according to ACT score. Furthermore, overweight or obesity is shown to be detrimental to lung function, characterized mainly by reduced FEV_1_ and FVC [13]. Physiological studies suggested that obesity decreased lung volume, which was associated with airway narrowing [52]. In our study, post-bronchodilator FEV_1_% pred was decreased in overweight or obese patients. In addition, overweight or obese patients tended to have more comorbidities. Obesity is associated with a range of asthma-related comorbidities, as it is reported to be associated with obstructive sleep apnea and gastroesophageal reflux that can affect asthma symptomatology [19, 53]. These were the potential causes that overweight or obese patients had poorer lung function and more asthma symptoms. It should be noted that the relationship between obesity and asthma is complex, influenced by many other factors including asthma-related comorbidities and some factors that produce obesity [54]. A study even showed that obesity does not increase the risk of asthma readmissions [54]. The effect of obesity on asthma control may need to be confirmed after expanding the sample size in the future.

Approximately 50% of the patients had fixed airway obstruction and more than three-quarters of them were non-smokers. Heavy smokers in our study were excluded to avoid misclassification of asthma with COPD. Fixed airway obstruction in older non-smoking asthmatic subjects may be related to increased lung compliance and loss of elastic recoil [55, 56]. We compared the characteristics of severe asthma patients with FAO to those without FAO. Patients with FAO were older, with longer duration of asthma, and lower pre-bronchodilator FEV_1_% pred than patients without FAO. Several studies found that FAO was associated with increased exacerbation rate [18, 57]and mortality [58, 59]. Consistent with this finding, we observed significantly difference in the number of exacerbations between patients with and without FAO. It should be noted that a study about the risk factors of FAO in older adults of asthma found that FAO is not independently associated with worse asthma control, quality of life, or exacerbations in older patients with asthma after controlling for confounding factors [60]. Therefore, we need to take into consideration of the age of FAO patients. For older patients with FAO, doctors should focus on other previously established risk factors rather than focus on maximizing lung function to improve care outcomes in this population.

Previous studies have confirmed the relationship between ACT score and lung function, exacerbations [61]. AQLQ score was affected by lung function, exacerbations, and BMI [62]. In our study, both of quality of life and asthma control were affected by the number of exacerbations and comorbidities in our study.

Our study has some limitations. First, all patients in this study were from one institution, indicating the potential for selection bias. Second, we only described baseline characteristics and lacked long-term follow-up data. Third, induced sputum is a valuable research tool for detecting airway inflammation and identifying asthma phenotypes. Due to lack of induced sputum data in our study, the patient’s asthma phenotype is not very clear.

In summary, this study performed a single-center retrospective analysis based on Chinese population in patients with severe asthma. Our findings contributed to improve our understanding of severe asthma in Chinese population and assisted us to learn the differences in clinical features of different phenotypes, which may lead to optimization of strategies to prevent and treat asthma.

## Conclusion

Severe asthma patients were more likely to be overweight or obese. The existence of comorbidities in severe asthma patients may result in more asthma symptoms and decreased quality of life. Patients with exacerbations or with overweight or obese phenotype were characterized by poorer lung function and worse asthma control. Patients with FAO phenotype tended to have more exacerbations. Both of quality of life and asthma control were affected by the number of exacerbations and comorbidities.

## Data Availability

The datasets generated and/or analyzed during the current study are not publicly available due to the fact that individual privacy could be compromised but are available from the corresponding author on reasonable request.

## Abbreviations

BMI: Body mass index
hx2: House dusts mix
mx2: Molds and yeasts mix
fx5: Food allergen mix
wx5: Weed pollen mix
tx4: Tree pollen mix
IgE: Immunoglobulin E
ICS: Inhaled corticosteroid
LABA: Long-acting beta agonist
LAMA: Long-acting muscarine antagonist
LTRA: Leukotriene receptor antagonist
OCS: Oral corticosteroid
FEV_1_: Forced expiratory volume in 1 s
FVC: Forced vital capacity
FENO: Fractional exhaled nitric oxide
ACT: Asthma control test
AQLQ: Asthma quality of life questionnaire
FAO: Fixed airway obstruction
